# Evaluating Knowledge, Self-Reported Confidence Levels, and Prescription Patterns among Dental Practitioners Regarding Analgesics in Dentistry: A Cross-Sectional Study

**DOI:** 10.3390/medicina60030467

**Published:** 2024-03-12

**Authors:** Marija Badrov, Antonija Tadin

**Affiliations:** 1 Department of Restorative Dental Medicine and Endodontics, Study of Dental Medicine, University of Split School of Medicine, 21000 Split, Croatia; mb91802@mefst.hr; 2 Department of Oral and Maxillofacial Surgery, Clinical Hospital Centre Split, 21000 Split, Croatia

**Keywords:** analgesics, dental pain, dentistry, knowledge, prescription

## Abstract

*Background and Objectives*: Dental pain is a common problem that often leads to unscheduled dental visits and requires a comprehensive understanding of analgesics, including their indications and contraindications. The aim of this study was to investigate dentists’ knowledge, self-reported confidence levels, and prescribing patterns of analgesics in dentistry. *Materials and Methods*: A nationwide cross-sectional online survey was conducted, resulting in 379 responses. Of these, 68.6% were general dentists, and 31.4% were specialists. The collected data included sociodemographic information, levels of knowledge, and prescription patterns. The survey questionnaire explored self-perceived practices, patient information during prescription, and guiding factors. Descriptive statistics and a generalized linear model for regression were used for data analysis. *Results*: Higher levels of knowledge were observed in specific contexts such as secondary/tertiary healthcare (*p* = 0.022), specialization in endodontics (*p* = 0.003), and a higher number of working hours with patients (*p* = 0.038). Conversely, increased self-confidence was observed among endodontists (*p* = 0.008), oral surgeons (*p* = 0.011), and dentists with more than 6 h of patient interaction (*p* ≤ 0.001). Orthodontists and prosthodontists demonstrated lower knowledge levels, while specialists in family dentistry exhibited lower self-confidence. Self-confidence and knowledge displayed a significant positive correlation (r = 0.039, *p* < 0.001). The most frequently prescribed medication was ibuprofen (97.9%), primarily for surgical (83.9%) and endodontic procedures (60.9%), with the main indications being pulpal (85.8%), periradicular (57.3%), and postoperative pain (40.1%). *Conclusions*: This study reveals significant knowledge and confidence gaps among dentists, including limited awareness of the efficacy of nonsteroidal anti-inflammatory drugs for odontogenic pain, a lack of time for effective counseling, and perceived deficits in pharmacology education. To address these issues, targeted educational interventions are recommended to improve analgesic prescribing practice, close knowledge gaps, and increase dentists’ confidence in more effective pain management.

## 1. Introduction

Effectively managing pain presents a major challenge in dentistry, as pain is a common reason for unplanned visits to the dentist [1]. Patients often come to the dental clinic complaining of pain and/or swelling, often due to odontogenic causes such as caries with or without pulp involvement. Most of these conditions can be successfully treated by conventional treatments such as fillings, root canal therapy, or extractions. In certain cases, the additional use of antibiotics and/or analgesics may be required alongside these interventions [2,3]. These medications are among the most frequently prescribed medications in dentistry. Consequently, dentists must demonstrate expertise in pain diagnosis and management and have a comprehensive understanding of the medications prescribed to patients [1,2,3].

Numerous studies have investigated analgesic knowledge and prescription trends in dentistry [3,4,5,6,7,8,9,10,11,12,13,14]. The primary reasons for prescribing analgesics were pain resulting from surgical procedures or endodontic conditions [8,10,12]. In particular, nonsteroidal anti-inflammatory drugs (NSAIDs), with a focus on ibuprofen, were prescribed as the most commonly prescribed medications [8,10,11]. The preferred initial drug choice for managing moderate to severe pain, such as pulpal or postsurgical pain, is typically administered at a dosage of 400–600 mg every six hours, with a maximum daily dose of 2400 mg [15]. Recent studies indicate that preemptive administration of analgesics before surgery or medical procedures is more effective in achieving superior pain control compared to their postoperative use [16]. While opioids are not typically the initial treatment option in dental care, they are often prescribed for severe pain that does not respond adequately to other medications. However, it is important to recognize that their prolonged use carries a significant risk of addiction [4]. According to research findings, when dealing with severe pain, the combined use of ibuprofen and paracetamol has been shown to be more effective for severe pain than the use of either medication alone. Moreover, this combination therapy has been found to outperform opioids in terms of effectiveness [2]. However, caution is required when prescribing NSAIDs due to their potential adverse effects on the gastrointestinal, cardiovascular, and renal systems. Furthermore, they should be avoided during pregnancy, particularly beyond 32 weeks of gestation, due to their association with the closure of the ductus arteriosus and impaired fetal circulation in utero [8].

Prescribing medication is an important aspect of medical practice aimed at curing disease, alleviating symptoms, and preventing future illness. This complicated task requires diagnostic skills, familiarity with commonly used medications, an understanding of the principles of clinical pharmacology, effective communication, and the ability to make decisions based on a thorough consideration of potential benefits and risks. The optimal choice aims to strike a balance between benefit and harm, taking into account both drug-related and patient-related aspects, while also considering limitations in terms of availability and cost [17]. Some specific groups of patients may require extra caution when taking these drugs [14]. The studies have shown that a significant proportion of dentists are not aware of these potential complications, nor do they follow current protocols when prescribing analgesics, including cross-sectional studies among Albanian [8], Tunisian [10], and Lebanese [14] dentists. Moreover, a concerning proportion of dentists fail to allocate adequate time for explaining medication administration to patients and providing proper instructions [7,8,12,14].

Analgesics have broad usage in the Republic of Croatia. Over the past two decades, there has been a consistent rise in both the overall cost and consumption of these medications in a country [18]. The prevalent oral diseases in Croatia in 2020 included dental caries and diseases of the pulp and periapical tissues, with dental fillings and treatment of oral soft tissue being the most common interventions [19]. Alongside antibiotics, analgesics rank as the most commonly prescribed drugs in dentistry in the country. NSAID prescriptions experienced an approximate 46% increase from 2014 to 2018. There has been a notable surge in the utilization of analgesics derived from propionic acid, notably ibuprofen and ketoprofen, while the usage of diclofenac demonstrated a statistically significant decrease [18,19,20,21]. Studies also indicated that Croatia has low opioid consumption rates, with tramadol being the most frequently prescribed narcotic [18,19].

Based on the literature reviewed, it was found that only a limited number of studies have investigated dentists’ awareness of analgesics and their prescribing patterns in dentistry [3,4,5,6,7,8,9,10,11,12,13,14]. These studies have the potential to provide valuable insight into identifying potential deficits in understanding and clinical use, paving the way for the development of tailored interventions aimed at improving standards of pain management in the dental setting. As far as we know, no studies have been conducted in Croatia on this specific topic. The aim of this study was to assess (1) knowledge of analgesics, (2) prescribing behavior, and (3) confidence in administering analgesics among dentists in Croatia. The null hypothesis was that there are no differences in knowledge, prescribing habits, or confidence in administering analgesics among dental practitioners in Croatia.

## 2. Materials and Methods

### 2.1. Study Design and Population

This cross-sectional study was conducted from 1 December 2023 to 15 January 2024 at the Department of Restorative Dentistry and Faculty of Endodontics, University of Split, Croatia. The data was collected via the Google survey tool (Google Forms, Google, Mountain View, CA, USA). The authors contacted the respondents via an available online e-mail, invited them to participate in the study, and sent them the link to the survey. Participation was voluntary, anonymous, and included consent, as participants were informed of this at the beginning of the survey. Reminder emails were sent, spaced two weeks apart, following the initial email invitation.

The study’s inclusion criteria encompassed dental practitioners in Croatia who demonstrated both the willingness and capability to engage in an online survey, along with a minimum of one year of clinical experience. The exclusion criteria included incomplete questionnaires and dental practitioners who were either retired or not actively engaged in clinical practice, as well as recently graduated dental students and dental residents in their first year.

The minimum necessary sample size (n = 351) was determined utilizing the Sample Size Calculator (Inc.RaoSoft^®^, Seattle, WA, USA), an online tool. This calculation was based on an estimated population of 3928 dental practitioners working in the Croatian health care system (consisting of 511 specialists and 3417 general dentists), an expected response rate of 50%, a confidence level of 95%, and a margin of error of 5% [22].

The study was approved by the Ethics Committee in accordance with the applicable guidelines and regulations, including compliance with the Declaration of Helsinki of the World Medical Association. The protocol of the study was approved by the Institutional Review Board, School of Medicine, University of Split, Croatia (Class: 003-08/23-03/0015, No.: 2181-198-03-04-23-0080). The study was conducted in accordance with the Helsinki Declaration of 1975, as revised in 2013. The study participants gave their informed consent to participate via the online interface.

### 2.2. Questionnaire

The questionnaire was developed and adapted from previous studies focused on analgesics in dental practice [3,4,5,6,7,8,9,10,11,12,13,14]. A working group, comprising two dental practitioners who are university professors specializing in endodontics, critically reviewed and assessed the questionnaire for content validity. Subsequently, the survey underwent a pilot test involving 30 dental practitioners to ensure its readability and understandability. Following the pilot testing, no revisions were made to the questionnaire. The pilot study also assessed the estimated time required to complete the questionnaire, concluding it to be approximately 15 min. Importantly, participants in the pilot survey were distinct from those included in the primary data collection.

The self-administered questionnaire consisted of 65 questions, divided into six sections. The initial section of the questionnaire comprised eight (Q1–Q8) demographic inquiries concerning dental practitioners. These questions encompassed gender, age, educational attainment, specialization, practice setting, years of experience in dental practice, and the average number of patients and number of working hours with patients per day.

The second part of the questionnaire focused on examining knowledge of analgesics (12 questions, Q9–Q20), where respondents were asked to choose between three options: “Yes”, “No” or “I do not know”. A scoring system was introduced for this questionnaire, where correct answers (“Yes”) were given a score of one and incorrect answers were given a score of zero. The cumulative score for each respondent, based on the number of correct answers, served as a quantitative measure to assess the individual’s level of knowledge. Respondents could achieve a maximum of 12 points in the part of the questionnaire used to test their level of knowledge.

In the third segment, dental practitioners encountered 15 questions (Q21–Q35), prompting them to assess their confidence level in administering analgesics. This evaluation utilized a five-level Likert scale (1—not confident, 2—slightly confident, 3—somewhat confident, 4—fairly confident, and 5—very confident). The total score was determined by summing the points acquired for each response, with a maximum score of 75 points indicating the highest level of confidence.

The fourth section consisted of 16 questions (Q36–Q51) aimed at assessing dental practitioners’ self-reported knowledge and experience concerning analgesics. These questions were designed to gauge their understanding of the use of analgesics in dentistry and to determine their self-reported confidence in prescribing such medications. The survey also delved into analgesic prescription patterns and explored challenges encountered in dental practice. Furthermore, it included five questions specifically addressing the medications and indications for which dental practitioners most frequently prescribe analgesics.

**Figure 1:**
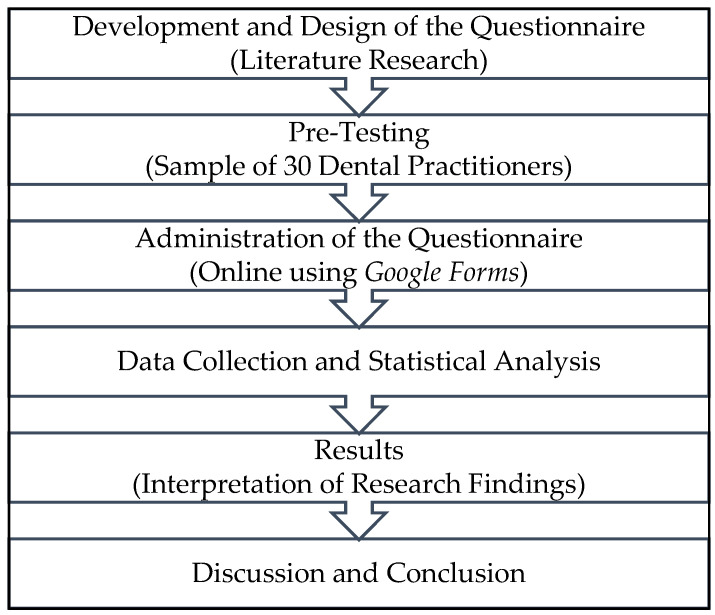
The flow chart of the survey methodology.

The fifth section, consisting of 7 queries (Q52–Q58), explored participants’ practices in prescribing analgesics, investigating elements such as rational prescription and consideration of general factors, among others. Meanwhile, the final section, comprising seven questions (Q59–Q65), concentrated on assessing the frequency of information provided to patients regarding the usage of analgesics. Figure 1 presents the flow chart of the questionnaire survey methodology.

### 2.3. Data Analysis

Statistical analysis was conducted using SPSS Statistics version 26.0 (IBM Corp., Armonk, NY, USA), and significance was assessed at a *p*-value of less than 0.05. The normality of the data was evaluated using the Kolmogorov–Smirnov test. Descriptive analysis was employed, presenting categorical variables as frequency and percentage. Given the non-normal distribution of the data, continuous variables were expressed as the median (interquartile range, IQR), while frequencies and percentages were used for categorical variables. A generalized linear model (GLM) analysis was performed to identify characteristics associated with knowledge and self-confidence level scores. The knowledge score (with a median of 7 points and observed values ranging below and above 7 points) and self-confidence level (with a median of 51 points and observed values below and above 51 points) served as dependent variables. Independent variables encompassed gender, age in years, education level, specialization, years in dental practice, number of patients per working day, experience of local or systemic complications, as well as dental practitioners’ self-reported knowledge and experience regarding analgesics. Spearman’s correlation was also employed to assess the association between sociodemographic factors and the levels of knowledge and self-confidence, as well as the correlation between knowledge and self-confidence levels themselves.

## 3. Results

A total of 379 respondents took part in the study, of whom 68.6% (n = 260) were general dentists (Table 1). The mean age of the respondents was 38.95 ± 10.16 (Md 37.00, IQR 30.00–47.00), and the mean working experience was 12.93 ± 10.26 years (Md 10.00, IQR 4.00–10.00). Respondents devoted an average of 6.49 ± 1.49 h per working day to patient care (Md 7.00, IQR 6.00–8.00) and cared for an average of 11.12 ± 4.63 patients per day (Md 10.00, IQR 8.00–15.00).

Among the sociodemographic characteristics investigated, factors such as gender, age, academic degree, years of clinical experience, and the average number of patients per day did not show a significant influence on the level of knowledge about the use of analgesics in dentistry and self-confidence in prescribing analgesics. However, a significantly higher level of knowledge was found in relation to the working environment (secondary and tertiary health care or dental school) (OR 1.031, 95% CI 0.151–1.911, *p* = 0.022), specialization in endodontics (OR 2.293, 95% CI 0.788–3.998, *p* = 0.003), and a higher number of working hours with patients (OR 0.501, 95% CI 0.028–0.975, *p* = 0.038). Higher self-confidence in prescribing analgesics was observed in dentists, especially endodontists (OR 1.649, 95% CI 0.434–2.831, *p* = 0.008), oral surgeons (OR 1.614, 95% CI 0.376–2.851, *p* = 0.011), and dentists who work with patients for more than 6 h per day (OR 0.961, 95% CI 0.470–1.451, *p* ≤ 0.001).

In terms of specialization, endodontists (8.88 ± 1.85) and oral surgeons (8.17 ± 2.31) demonstrated the highest levels of knowledge, while orthodontists (3.17 ± 3.02) and prosthodontics (5.56 ± 2.65) exhibited the lowest levels of knowledge in this context. Endodontists and oral surgeons showed the highest self-confidence in prescribing analgesics, with scores of 58.29 ± 9.55 and 57.92 ± 16.28, respectively. In contrast, specialists in family dentistry had the lowest self-confidence, with a value of 30.00 ± 13.22.

Table 2 illustrates the frequency of correct and incorrect answers to questions on the use of analgesics in dentistry. The overall knowledge score of all respondents averaged 6.82 ± 2.58 (Md 7.00, IQR 5.00–9.00, min 0, max 12) out of a possible 12 points. The majority (54.9%, n = 208) of respondents had a level of knowledge at or above the median. Notably, two respondents (0.5%) did not provide a single correct answer, while nine (2.4%) answered all questions correctly. In terms of specific questions, more than 90% (n = 343) of respondents answered the question “Long-term use of opioids can lead to addiction.” correctly. However, only 29.6% (n = 112) of respondents knew the correct answer to the question, “In most cases, NSAID analgesics are more effective than opioids in the treatment of odontogenic pain”.

Table 3 shows the self-confidence of dentists when prescribing analgesics. The mean self-confidence of all respondents was 49.99 ± 12.81 (Md 51.00, IQR 44.00–59.00, min. 15, max. 74) with a maximum score of 75. Only four respondents (1.1%) reported uncertainty in prescribing analgesics across all 15 questions. Dentists showed the highest level of confidence in dosing and administering analgesics. Conversely, their confidence was lowest when prescribing analgesics for patients with renal, metabolic, and respiratory diseases.

Table 4 presents data on dental practitioners’ self-reported knowledge and experience of prescribing analgesics. Those who self-rated their knowledge as good had higher levels of knowledge (OR 2.469, 95% CI 0.331–4.608, *p* = 0.024) and confidence (OR 1.793, 95% CI 0.192–3.393, *p* = 0.028). Similar results were observed among respondents who felt that they had received sufficient information about analgesics during and after their studies (OR 0.736, 95% CI 0.233–1.239, *p* = 0.004 and OR 0.590, 95% CI 0.065–1.116, *p* = 0.028, respectively). In addition, respondents who experienced complications in their patients after analgesic prescription showed a significant difference in self-confidence (OR 1.594, 95% CI 0.735–2.452, *p* ≤ 0.001). 

Table 5 presents data on the utilization of analgesics, encompassing the type of analgesics, reasons for their use, sources of knowledge, and obstacles encountered in their use and knowledge. Predominantly, respondents commonly prescribe ibuprofen (n = 371, 97.9%) and paracetamol (n = 195, 51.5%). These medications are frequently administered during surgical (n = 318, 83.9%) and endodontic procedures (n = 231, 60.9%), primarily for pulpal (n = 325, 85.8%), periradicular pain (n = 217, 57.3%), and postoperative pain (n = 152, 40.1%). The most prominent obstacle to conducting medication counseling in daily patient interactions, as indicated by respondents, is the lack of time (n = 194, 51.2%), followed closely by the lack of education in the field of pharmacology (n = 189, 49.9%).

**Figure 2:**
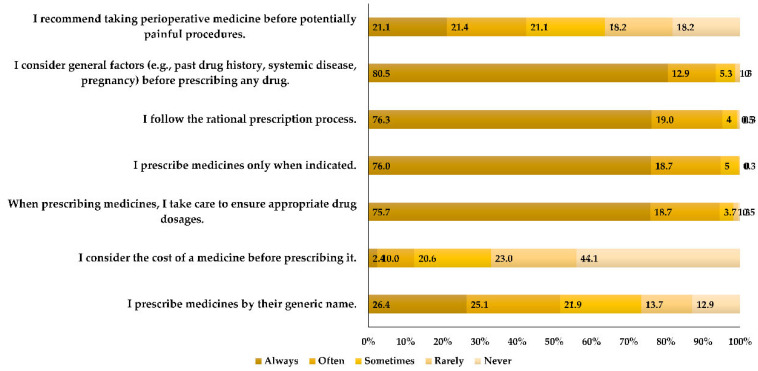
Participant practices in prescribing analgesics and considerations in medicinal decision-making (Q53–58).

Figure 2 demonstrates prescribing habits among dentists. Most respondents exhibited favorable practices in prescribing medications, including always considering the general factors of the patient (n = 305, 80.5%), following rational prescription processes (n = 289, 76.3%), prescribing medicines only when indicated (n = 288, 76.0%), and ensuring appropriate drug dosages (n = 287, 75.7%). Conversely, nearly half of the participants (n = 167, 44.1%) have never taken into account the cost of the medication.

**Figure 3:**
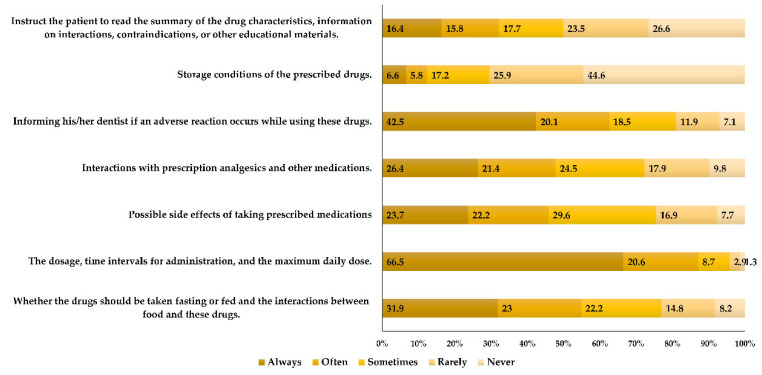
The frequency of providing instructions to patients before administering analgesics (Q59–65).

Figure 3 shows the communication and giving of information to the patients. The most common information always provided to patients was the dosage, time intervals for administration, and the maximum daily dose (n = 252, 66.5%). Only 26.4% alert their patients to possible interactions with other medications, while merely 23.7% of dentists provide warnings to their patients regarding potential side effects. Slightly less than half of the respondents (n = 169, 44.6%) never provide instructions to their patients regarding the proper storage of medication.

Spearman’s analysis revealed a significant positive correlation of knowledge with self-confidence level (r = 0.039, *p* < 0.001) and a negative correlation with the age of the respondents (r = −0.117, *p* = 0.023). Furthermore, self-confidence level showed significant positive correlations with knowledge (r = 0.039, *p* < 0.001), age (r = 0.114, *p* = 0.028), years of clinical working experience (r = 0.114, *p* = 0.005), and working hours with patients per day (r = 0.141, *p* = 0.006).

## 4. Discussion

Effectively addressing pain presents a significant challenge in the field of dentistry, with the occurrence of pain being one of the most common reasons for unscheduled visits to the dentist. In addition to antibiotics, analgesics constitute the second most commonly prescribed category of medications by dentists. Therefore, it is imperative for dentists to possess comprehensive knowledge of analgesics, particularly understanding their indications and contraindications [1,2]. The aim of this study was to assess dentists’ knowledge, prescribing practices, and confidence in prescribing analgesics in clinical practice. The aim was to provide a solid foundation that can be used to improve their understanding and attitude towards this topic. A review of the literature revealed that few studies have addressed dentists’ knowledge of analgesics and their prescribing patterns in dentistry [3,4,5,6,7,8,9,10,11,12,13,14]. To date, no study on this topic has been conducted in Croatia. Therefore, there is a significant information gap on this topic, especially regarding the correlation between the studied aspects, including the understanding of analgesics in dentistry and confidence in dealing with different patient profiles.

The null hypothesis of this study was that there were no differences in knowledge, prescribing patterns, or confidence levels when it came to administering analgesics among dental practitioners in Croatia. No difference was found among the respondents in knowledge of self-confidence depending on academic qualifications, but there was a difference among individual specializations compared to general dentists. Also, no differences were found depending on work experience and the number of patients treated per working day.

In this study, dentists displayed suboptimal knowledge and self-confidence in prescribing analgesics in dentistry. A significant correlation existed between knowledge and self-reported confidence levels, with younger respondents exhibiting higher knowledge levels and the elderly showing higher self-reported confidence. Effective decision-making in clinical settings necessitates both knowledge acquisition and confidence development. Mismatched confidence and competence can lead to patient harm, underscoring the importance of aligning confidence levels with training and clinical complexity [23,24]. The self-reported confidence level significantly correlates with longer working hours spent with patients per day. Additionally, respondents working in secondary or tertiary dental care demonstrated better knowledge. These findings were partially corroborated by a study in Guangzhou, which investigated analgesic and antibiotic prescription patterns among dentists. It revealed that experienced dentists lacking postgraduate courses tended to prescribe inappropriate antibiotics and analgesics more frequently compared to newly trained and postgraduate dentists [7]. In the present study, it is noteworthy that individuals working in secondary and tertiary healthcare or dental schools, specializing in endodontics, and those with a greater number of working hours with patients exhibited a superior understanding of analgesics in dentistry. Similarly, significant associations were observed between self-confidence levels in prescribing analgesics and professional characteristics such as specialization in endodontics, oral surgery, and an extended duration of patient interaction exceeding 6 h per day. Considering that dentists frequently encounter oral conditions characterized by inflammatory pathologies, notably pain-associated issues such as pulpitis and periapical periodontitis, alongside various oral surgical procedures, it is understandable that specialists in these domains demonstrate elevated knowledge and confidence in this specific subject matter. In the realm of oral surgery and endodontics, systemic medication is occasionally recommended as an adjunct to treatment, targeting pain, inflammation, and infection control. Antibiotics, analgesics, and anti-inflammatory drugs are commonly prescribed medications in the practice of maxillofacial surgery [6,25,26]. On the other hand, orthodontists and prosthodontists exhibited the lowest levels of knowledge in this context. These findings may raise concerns for orthodontists, given that NSAIDs are widely utilized for pain management in orthodontic care, yet debates persist regarding their impact on tooth movement, leading to varying usage practices. Prioritizing pain control is paramount in orthodontic treatment. Therefore, orthodontic specialists need to enhance their understanding of analgesics [27]. A cross-sectional study conducted in Lebanon on the assessment of drug-prescribing perception and practice among dental care providers has also shown similar results regarding higher confidence level, and specialties in oral surgery and endodontics. On the other hand, specialists in periodontology in that study had the lowest odds of self-confidence in prescribing drugs [14].

With regard to specific questions related to analgesics, the one with the highest accuracy was related to the correlation between prolonged opioid usage and the risk of addiction (n = 343, 90.5%). A study among Indian dentists has found a smaller (72%) proportion of their participants supporting these statements [4]. In the present study, however, the question with the lowest number of correct answers pertained to the interaction of NSAIDs with the antiplatelet effect of acetylsalicylic acid, with only 21.9% (n = 83) providing accurate responses. It is important to emphasize that dentists must be aware of these potential interactions. Given that various dental procedures, including tooth extractions and other operative treatments, commonly induce pain and inflammation, NSAIDs are often prescribed for symptom management. However, when patients are concurrently taking ASA for cardiovascular protection, dentists must assess the risk of drug interactions and contemplate alternative pain management strategies as necessary [28,29].

For moderate to severe pain, it is recommended to administer a combination of ibuprofen and paracetamol, as this combination is more effective than the individual medications. In addition, this combination is more effective compared to opioids [2]. In the present study, 64.9% (n = 246) of the respondents were aware of the synergistic effect of these drugs, while only 29.6% (n = 112) knew that NSAID analgesics are more effective than opioids in the treatment of odontogenic pain. In other studies, e.g., among dentists in India [4] and the USA [5], a significantly higher proportion of participants agreed with these findings. The administration of NSAIDs is discouraged after the 30th week of gestation due to the potential risk of fetal pulmonary hypertension and premature closure of the ductus arteriosus [8]. Just over half of the respondents in this study (n = 210, 55.4%) were aware of this fact, which constituted a markedly higher percentage compared to dentists in the study from Albania [8]. Recent studies support the idea that administering analgesics preemptively before surgery or a medical procedure is more effective in achieving superior pain control compared to their postoperative use when anticipating pain after the procedure [16]. This finding was corroborated by just over half (n = 208, 54.9%) of the dentists participating in this study. A corresponding observation was noted in an Iranian study involving dentists managing post-endodontic pain [11].

Dentists routinely engage in medication prescribing within their professional practice. Factors such as education, clinical experience, support systems, familiarity with drugs, and patient-related variables collectively influence doctors’ self-confidence in prescribing medications [30]. In this study, dentists demonstrated the highest level of confidence in dosing and administering analgesics, with 76.5% of respondents indicating that they were either fairly or very confident in this regard. On the other hand, their confidence was notably diminished when prescribing analgesics for patients with renal (31.1%), metabolic (29.3%), and respiratory diseases (30.1%). Dentists involved in a cross-sectional study from Lebanon showed similar confidence levels when prescribing analgesics to patients with respiratory diseases, while their confidence was much higher for patients with metabolic diseases (44.5%) [14]. Conversely, there was a noticeable disparity in confidence levels when dealing with patients diagnosed with malignant diseases, and this study uncovered a slight elevation in confidence specifically linked to this aspect compared to the Lebanese study.

According to the study on the national consumption of opioid and nonopioid analgesics in Croatia in the period from 2007 to 2013, the country exhibited low opioid consumption [18]. This study has demonstrated that opioids are also not extensively utilized in the field of dentistry, while only 10.6% (n = 40) of dentists prescribe these drugs. Opioids were notably more frequently prescribed in studies conducted in India [4] and the USA [5]. As per studies of medication prescribing practices in Croatian dental offices and their contribution to national consumption, ibuprofen was the first-choice analgesic in dental medicine [19,20,21]. In this study, this medication emerged as the most commonly prescribed analgesic (97.9%), followed by paracetamol in second place (51.5%). Ibuprofen was also preferred in studies among dentists from Albania [8], Tunisia [10], and Iran [11]. Meanwhile, other studies have revealed varying preferences in the selection of analgesics, including diclofenac, by Indian [6] and Chinese [7] dentists. A study involving Albanian dentists [8] similarly indicated widespread usage of ketoprofen and paracetamol, whereas a Turkish study [12] revealed a preference among their practitioners for naproxen.

The primary procedures for which analgesics were prescribed, as reported by the participating dentists in this study, were surgical (83.9%) and endodontic (60.9%), with pulpal (85.8%), periradicular (57.3%), and postoperative pain (40.1%) being the most common indications. Consistent results were found in other studies, e.g., in research on dentists’ knowledge of over-the-counter NSAIDs [8], in a survey of Tunisian dentists’ prescribing practices [10], and in a study of dentists in Istanbul [12].

The predominant sources of education on this specific topic, as reported by the practitioners, included dental studies (n = 250, 66.0%) and continuous education activities, such as seminars and congresses (n = 190, 50.1%). The study of Albanian dentists revealed that a minimal proportion of their practitioners (6.89%) engaged in seminars following their completion of dental studies [8]. Furthermore, an Indian study identified the internet as the primary access point for information [13], while research among Lebanese dentists highlighted the role of clinical pharmacists [14]. The greatest obstacles to not conducting medication counseling in daily work with patients were lack of time (51.2%) and lack of pharmacology education (49.9%). Moreover, nearly three-quarters (72.3%) of the participants in this study typically spent three minutes or less on the prescription and explanation of analgesic use to their patients. On the other hand, dentists in the Lebanese study, on average, dedicated more time than participants in this study [14].

The majority of respondents showed positive practices when prescribing medication. These included consistent consideration of general patient factors (n = 305, 80.5%), adherence to rational prescribing processes (n = 289, 76.3%), prescribing medications only when needed (n = 288, 76.0%), and confirming appropriate medication dosage (n = 287, 75.7%). However, a study of dentists’ practices and knowledge of analgesic and antibiotic prescribing in Saudi Arabia found a significantly higher proportion of participants engaging in this behavior [9]. A study examining the nationwide consumption of opioid and non-opioid analgesics in Croatia from 2007 to 2013 found a significant increase in the total costs associated with these medications in the country [18]. In spite of that, nearly half of the dentists (n = 167, 44.1%) in this study have never taken into account the cost of the medication, while more than a half of Saudi Arabian dentists (58.73%) have never considered this issue [9].

This study revealed suboptimal communication and the insufficient provision of clear instructions regarding the administration of analgesics to patients. Most of the respondents in this study (n = 252, 66.5%) always make sure to inform their patients on the dosage, time intervals for administration, and the maximum daily dose. Only 26.4% of dentists inform their patients about potential interactions with other medications, and a mere 23.7% provide warnings about possible side effects. Merely 6.6% of them provide guidance to patients on the storage conditions of the medication. A study of analgesic prescription patterns in the management of dental pain among dentists in Istanbul has also demonstrated an even greater lack of this information [12]. Conversely, certain studies have demonstrated notably improved behavior regarding communication with patients. Nearly all (93.02%) of the dentists in Saudi Arabia [9] and 80.1% of those in Guangzhou [7] stated that they consistently take the time to educate patients about medication usage, including discussing potential side effects. Research indicates that patients frequently do not receive the desired information about their medications. Despite evidence emphasizing that understanding medications and being satisfied with that understanding are significant predictors of medication adherence, this gap persists. A study investigating the viewpoints of general medicine inpatients underscored the necessity of effectively providing information to patients. Key themes encompassed autonomy, nurturing relationships, access to information, communication, and minimal informational requirements [31].

This study is subject to several limitations that warrant acknowledgment. Firstly, it depended on an online cross-sectional survey, thereby relying on self-reported knowledge and practices for the collected data. Additionally, there is a possibility that individuals who required further knowledge about the study topic opted not to participate, potentially affecting the generalizability of the findings. Another constraint of the study is the limited sample size. The study also revealed a potential selection bias concerning the participation of specialists, with a small proportion represented in oral medicine and family dentistry. Lastly, the study participants displayed an imbalanced gender distribution, with a greater number of women participating compared to men. Given these limitations, it is crucial to approach the interpretation of the study findings with caution and acknowledge the potential biases that might have impacted the results. Dentists, as healthcare professionals, frequently prescribe analgesics to manage pain and inflammation in their patients. Therefore, possessing sufficient knowledge of analgesics, including their indications, contraindications, and administration to high-risk patients, is imperative for the practice of dentistry and the safety of patients [7,8,9,10]. The responsibility for dentists’ knowledge of analgesics and their application primarily falls upon the dental education institution. After dental school, ongoing education significantly contributes to knowledge retention. However, recently graduated dentists in this study exhibited notably elevated levels of knowledge, whereas their older counterparts displayed higher levels of self-confidence in prescribing analgesics despite lower knowledge levels. Therefore, regulatory bodies, particularly the dental chamber responsible for their education, should enhance their efforts in organizing lectures and workshops addressing topics such as analgesics, along with their rational prescribing patterns and associated potential risks. Furthermore, it would be advantageous to implement a greater emphasis on clinically oriented pharmacology courses at dental schools while also encouraging collaboration with pharmacists.

## 5. Conclusions

The results of the study show a notable deficit in the respondents’ understanding of analgesics and confidence levels, particularly in relation to scenarios involving high-risk patients. Differences in knowledge were found among secondary and tertiary healthcare workers, particularly among endodontic and oral surgery specialists, who demonstrated higher levels of knowledge and confidence. Key challenges identified included a limited understanding of the efficacy of NSAIDs for odontogenic pain, barriers to effective medication counseling due to time constraints, and perceived deficits in pharmacology education. The study advocates tailored educational interventions aimed at improving analgesic prescribing practices in the field of dentistry.

## Figures and Tables

**Table 1:** Demographic and professional predictors for knowledge on the use of analgesics in dentistry and self-reported analgesics prescribing confidence.

Characteristics		n (%)	Knowledge Score		Self-Confidence Level	
			OR (95% CI)	*p*-Values	OR (95% CI)	*p*-Values
Q1. Gender	Man	95 (25.1)	Reference		Reference	
	Woman	284 (74.9)	0.335 (−0.178–0.849)	0.201	−0.402 (−0.938–0.134)	0.142
Q2. Age group (years)	≤30	112 (29.6)	Reference		Reference	
	31–40	115 (30.3)	−0.403 (−1.025–0.212)	0.198	0.179 (−0.449–0.806)	0.577
	41–50	89 (23.5)	−0.533 (−1.605–0.538)	0.329	0.086 (−1.018–1.190)	0.879
	≥51	63 (16.6)	−0.204 (−1.331–0.902)	0.717	0.287 (−0.860–1.434)	0.624
Q3. Academic qualification	DMD	277 (73.1)	Reference		Reference	
	MSc/PhD	102 (26.9)	−0.146 (−1.039–0.748)	0.749	−0.142 (−0.857–0.572)	0.696
Q4. Dental specialty	General dentist	260 (68.6)	Reference		Reference	
	Endodontics	24 (6.3)	2.293 (0.788–3.998)	0.003 *	1.649 (0.434–2.831)	0.008 *
	Oral surgery	24 (6.3)	0.790 (−0.244–1.824)	0.134	1.614 (0.376–2.851)	0.011 *
	Oral medicine	6 (1.6)	0.480 (−1.525–2.485)	0.639	0.113 (−1.907–2.132)	0.914
	Pediatric dentistry	17 (4.5)	0.109 (−1.035–1.253)	0.852	0.393 (−0.778–1.570)	0.508
	Orthodontics	14 (3.7)	−0.663 (−2.028–0.702)	0.341	0.757 (−0.626–1.114)	0.283
	Periodontics	15 (4.0)	0.568 (−0.730–1.867)	0.391	0.101 (−1.215–1.427)	0.875
	Prosthodontics	16 (4.2)	−0.355 (−1.600–0.890)	0.578	−0.620 (−1.872–0.631)	0.331
	Family dentistry	3 (0.8)	0.524 (−2.039–3.068)	0.689	−22.970 (−84,890.519–84,844.578)	1.000
Q5. Practice setting	Private practice	117 (30.9)	Reference		Reference	
	Health center	200 (52.8)	0.514 (−0.022–1.050)	0.060	−0.208 (−0.755–0.339)	0.458
	Secondary/tertiary care	62 (16.4)	1.031 (0.151–1.911)	0.022 *	0.917 (0.000–1.834)	0.050 *
Q6. Clinical working experience	≤10 years	192 (50.7)	Reference			
	>10 years	187 (49.3)	−0.146 (−1.039–0.748)	0.749	0.026 (−0.900–0.956)	0.956
Q7. Working hours with patients per day	≤6 h	184 (48.5)	Reference		Reference	
	>6 h	195 (51.5)	0.501 (0.028–0.975)	0.038 *	0.961 (0.470–1.451)	≤0.001 *
Q8. Number of patients per day	≤10 patients	217 (57.3)	Reference		Reference	
	>10 patients	162 (42.7)	0.106 (−0.374–0.586)	0.665	0.425 (−0.069–0.920)	0.092

Data are presented as numbers (percentages). The reference knowledge category is “low”. OR, odds ratio; 95% CI, 95% confidence interval. * *p* ≤ 0.05.

**Table 2:** The frequency distribution (%) of responses from dentists regarding questions on the use of analgesics in dentistry as part of a knowledge test.

Question	Answer	n (%)
Q9. Long-term use of opioids can lead to addiction.	Yes	343 (90.5)
	No	4 (1.1)
	I do not know	32 (8.4)
Q10. The use of NSAIDs is contraindicated after the 30th week of gestation due to the risk of fetal pulmonary hypertension and premature closure of the ductus arteriosus.	Yes	210 (55.4)
	No	21 (5.5)
	I do not know	148 (39.1)
Q11. The maximum recommended daily dose of paracetamol for healthy adults is 4000 milligrams (mg).	Yes	243 (64.1)
	No	69 (18.2)
	I do not know	67 (17.7)
Q12. The maximum recommended daily dose of acetylsalicylic acid for healthy adults is 4000 milligrams (mg).	Yes	114 (30.1)
	No	103 (27.2)
	I do not know	162 (42.7)
Q13. The maximum recommended analgesic daily dose of ibuprofen for healthy adults is 2400 milligrams (mg).	Yes	241 (63.6)
	No	76 (20.1)
	I do not know	62 (16.4)
Q14. Paracetamol (acetaminophen) is considered safe for use during both pregnancy and breastfeeding.	Yes	321 (84.7)
	No	29 (7.7)
	I do not know	29 (7.7)
Q15. Paracetamol (acetaminophen) is known to act synergistically with NSAIDs and opioids, enhancing their effectiveness when used together.	Yes	246 (64.9)
	No	34 (9.0)
	I do not know	99 (26.1)
Q16 Ibuprofen and naproxen are nonsteroidal anti-inflammatory drugs (NSAIDs) that may interfere with the antiplatelet effect of acetylsalicylic acid (aspirin).	Yes	83 (21.9)
	No	90 (23.7)
	I do not know	206 (54.4)
Q17. In the treatment of moderate pain in adults, when there are no contraindications for NSAID use, it is common to prescribe ibuprofen at doses of 400–600 mg every 4 to 6 h or naproxen at a dose of 500 mg (or naproxen sodium at 550 mg) every 12 h.	Yes	304 (80.2)
	No	33 (8.7)
	I do not know	42 (11.1)
Q18. In adult patients with a contraindication for NSAID use, it is common to prescribe acetaminophen at a dose of 1000 mg every 6 h for the treatment of moderate pain.	Yes	153 (40.4)
	No	52 (13.7)
	I do not know	174 (45.9)
Q19. The use of analgesics before surgery or a medical procedure is more effective than using them after the surgery or procedure to achieve better pain control when anticipating postoperative pain.	Yes	208 (54.9)
	No	90 (23.7)
	I do not know	81 (21.4)
Q20. In most cases, NSAID analgesics are more effective than opioids in treating odontogenic pain.	Yes	112 (29.6)
	No	111 (29.3)
	I do not know	156 (41.2)

Data are presented as numbers and percentages.

**Table 3:** The frequency distribution (%) of responses from dental practitioners concerning self-reported confidence levels in prescribing analgesics (Q21–35).

Characteristic	Self-Reported Confidence Level n (%)				
	Very Confident	Fairly Confident	Somewhat Confident	Slightly Confident	Not Confident
Q21. Dosage and drug administration	91 (24.0)	199 (52.5)	62 (16.4)	19 (5.0)	8 (2.1)
Q22. Drug and drug interaction	23 (6.1)	91 (24.0)	151 (39.8)	81 (21.4)	33 (8.7)
Q23. Drug and food interaction	27 (8.2)	70 (18.5)	142 (37.5)	94 (24.8)	46 (12.1)
Q24. Side effects	31 (8.2)	114 (30.1)	149 (39.3)	64 (16.9)	21 (5.5)
Q25. Contraindications	64 (16.9)	131 (34.6)	118 (31.1)	50 (13.2)	16 (4.2)
Q26. Pregnancy	123 (32.5)	145 (38.3)	66 (17.4)	28 (7.4)	17 (4.5)
Q27. Children	107 (28.2)	130 (34.3)	90 (23.7)	38 (10.0)	14 (3.7)
Q28. Breastfeeding	97 (25.6)	147 (38.8)	80 (21.1)	33 (8.7)	22 (5.8)
Q29. Geriatric patients	55 (14.5)	135 (35.6)	121 (31.9)	49 (12.9)	19 (5.0)
Q30. Patients with malignant diseases	56 (14.8)	75 (19.8)	136 (35.9)	78 (20.6)	34 (9.0)
Q31. Hematological patients	62 (16.4)	97 (25.6)	129 (34.0)	65 (17.2)	26 (6.9)
Q32. Gastrointestinal patients	52 (13.7)	103 (27.2)	146 (38.5)	49 (12.9)	29 (7.7)
Q33. Nephrological patients	47 (12.4)	71 (18.7)	148 (39.1)	75 (19.8)	38 (10.0)
Q34. Endocrine/metabolic patients	41 (10.8)	70 (18.5)	146 (38.5)	79 (20.8)	43 (11.3)
Q35. Respiratory patients	40 (10.6)	74 (19.5)	141 (37.2)	83 (21.9)	41 (10.8)

Data are presented as numbers and percentages.

**Table 4:** Dental practitioners’ self-reported knowledge and experience regarding analgesics as predictors of knowledge on the use of analgesics in dentistry and self-reported confidence in prescribing analgesics.

Characteristics		n (%)	Knowledge Score		Self-Confidence Level	
			OR (95% CI)	*p*-Values	OR (95% CI)	*p*-Values
Q36. Self-assessment of personal knowledge about analgesics	Poor	9 (2.4)	Reference		Reference	
	Moderate	206 (54.4)	1.697 (−0.434–3.827)	0.118	0.424 (−1.151–2.000)	0.579
	Good	164 (43.3)	2.469 (0.331–4.608)	0.024 *	1.793 (0.192–3.393)	0.028 *
Q37. Perceived sufficient level of education on analgesics during dental graduate and postgraduate studies	No	124 (32.7)	Reference		Reference	
	Yes	255 (67.3)	0.736 (0.233–1.239)	0.004 *	0.590 (0.065–1.116)	0.028 *
Q38. Interested in further education on the topic of analgesics	No	97 (25.6)	Reference		Reference	
	Yes	282 (74.4)	0.458 (−0.066–0.981)	0.087	0.075 (−0.478–0.628)	0.790
Q39. Average time spent on prescribing and explaining the use of analgesics to patients	≤3 min	274 (72.3)	Reference		Reference	
	>3 min	105 (27.7)	−0.043 (−0.548–0.462)	0.867	0.470 (−0.061–1.001)	0.083
Q40. Experienced complications following the administration of analgesics	No	334 (88.1)	Reference		Reference	
	Yes	45 (11.9)	−0.138 (−0.836–0.560)	0.669	1.594 (0.735–2.452)	≤0.001 *
Q41. Prescription of opioid analgesics in clinical practice	No	339 (89.4)	Reference		Reference	
	Yes	40 (10.6)	0.659 (−0.236–1.553)	0.149	0.285 (−0.662–1.233)	0.555
Q42. Average number of prescribed opioid analgesics per month	0	313 (82.6)	Reference		Reference	
	1–5	53 (14.0)	−0.204 (−0.966–0.558)	0.600	−0.324 (−1.656–1.009)	0.634
	>5	13 (3.4%)	0.565 (−0.676–1.807)	0.327	−0.323 (−1.180–0.434)	0.365
Q43. Prescription of NSAID analgesics in clinical practice	No	25 (6.6)	Reference		Reference	
	Yes	354 (93.4)	−0.135 (−1.596–1.326)	0.856	0.136 (−1.424–1.695)	0.865
Q44. Average number of prescribed NSAID analgesics per month	0	20 (5.3)	Reference		Reference	
	1–5	155 (40.9)	1.174 (−0.534–2.882)	0.178	−0.420 (−2.205–1.365)	0.645
	>5	204 (53.8)	1.396 (−0.301–3.092)	0.107	0.600 (−1.160–2.360)	0.504
Q45. Frequency of patients presenting with acute pain per week	≤5	274 (72.3)	Reference		Reference	
	>5	105 (27.7)	0.349 (−0.164–0.862)	0.182	−0.140 (−0.674–0.395)	0.609
Q46. Frequency of patients presenting with chronic pain per week	≤5	325 (85.8)	Reference		Reference	
	>5	54 (14.2)	−0.153 (−0.798–0.492)	0.642	−0.134 (−0.824–0.556)	0.704

Data are presented as numbers (percentages). The reference knowledge category is “low”. OR, odds ratio; 95% CI, 95% confidence interval. * *p* ≤ 0.05.

**Table 5:** Analgesic prescription patterns and challenges in dental practice.

Characteristics		n (%)
Q47. Most commonly prescribed analgesics *	Ketoprofen	81 (21.4)
	Ibuprofen	371 (97.9)
	Acetylsalicylic acid	9 (2.4)
	Diclofenac	60 (15.8)
	Paracetamol	195 (51.5)
	Ketorolac	4 (1.1)
	Hydrocodone	1 (0.3)
	Oxycodone	16 (4.2)
	Codeine	8 (2.1)
	Tramadol	13 (3.4)
	Other	15 (4.0)
Q48. Most common dental procedures associated with administering analgesics *	Restorative	29 (7.7)
	Periodontal	89 (23.5)
	Surgical	318 (83.9)
	Orthodontic	15 (4.0)
	Implant	135 (35.6)
	TMJ	91 (24.0)
	Endodontic	231 (60.9)
	Other	19 (5.0)
Q49. Main source of analgesics information *	Dental School	250 (66.0)
	Seminars and congresses	190 (50.1)
	Colleagues	157 (41.4)
	Clinical pharmacologists	45 (11.9)
	Pharmacists	90 (23.7)
	Internet	186 (49.1)
	Other	55 (14.5)
Q50. The most common types of pain for which patients seek help/intervention and analgesic prescriptions *	Dentin pain	132 (34.8)
	Pulpal pain	325 (85.8)
	Periradicular pain	217 (57.3)
	Periodontal pain	127 (33.5)
	Alveolar osteitis	130 (34.3)
	TMJ pain	61 (16.1)
	Pericoronitis	140 (36.9)
	Mouth ulcers	38 (10.0)
	Bruxism	35 (9.2)
	Dental trauma	55 (14.5)
	Sinusitis	23 (6.1)
	Postoperative pain	152 (40.1)
	Salivary glands pathology	12 (3.2)
	Stomatitis	26 (6.9)
Q51. Most significant obstacle to conducting medication counseling in my daily work with patients *	Not conducting counseling	47 (12.4)
	Lack of time	194 (51.2)
	Lack of pharmacology education	189 (49.9)
	Lack of rational drug administration knowledge	99 (26.1)
	Answering only if patients ask	74 (19.5)
	Other	49 (12.9)

Data are presented as numbers and percentages. * indicates a Multiple-choice question.

## Data Availability

Data supporting the findings of this study are available on request.
